# Rotavirus Vaccine Coverage and Surveillance of Adverse Events Following Immunization in a Birth Cohort of Children (2017–2023) in Suzhou, China

**DOI:** 10.3390/vaccines14020139

**Published:** 2026-01-29

**Authors:** Jinling Gao, Kunpeng Zhu, Lin Luan, Benfeng Zheng, Juan Xu, Yiheng Zhu, Xianquan Fan, Haitao Wang, Na Liu

**Affiliations:** 1 Suzhou Center for Disease Control and Prevention, Suzhou 215000, China; gaojinling_ss@163.com (J.G.);; 2 Suzhou Municipal Health Commission, Suzhou 215000, China

**Keywords:** rotavirus vaccine, rotavirus gastroenteritis, vaccination, adverse events following immunization (AEFIs)

## Abstract

**Objectives**: To analyze the changes in the proportion of the rotavirus vaccine among children born in the 2017–2023 cohort and to assess the current status of rotavirus vaccination coverage in Suzhou, China. To monitor adverse events following immunization (AEFIs) so as to provide data for scientific guidance regarding the rotavirus vaccine. **Methods**: The basic information of children born between 1 January 2017 and 31 December 2023 in Suzhou and information regarding rotavirus vaccination were collected through the child module of Jiangsu Province Vaccination Integrated Service Management Information System. Information on AEFI case reports was collected from the AEFI monitoring system of the China Information System for Disease Control and Prevention. Descriptive epidemiological methods were used to analyze the rotavirus vaccine characteristics and AEFI classification, and the Mann–Whitney U test was used for comparative analysis. **Results**: The proportion of children born in the 2017–2023 cohort who received the first dose of the rotavirus vaccine was 14.65%. The reassortant rotavirus vaccine, live, oral, pentavalent (RV5) proportion gradually increased, and the vaccine proportion of children in the 2023 birth cohort reached the highest. The peak age for rotavirus vaccination was between 2 and 8 months. A total of 49,507 children (99.88%) received the first dose of RV5 at the age of 6–12 weeks in this birth cohort, and there was a statistically significant difference in the median duration of the first dose of RV5 among children of different age groups (*p* < 0.001). A total of 89 cases of AEFIs were reported, and the reported incidence of AEFIs was 3.47/10,000 doses. Among them, 86 cases of general reactions were reported, with a reported incidence of 3.35/10,000 doses, and three cases of abnormal reactions were reported, with a reported incidence of 0.12/10,000 doses. **Conclusions**: The rotavirus vaccine proportion of children born in Suzhou from 2017 to 2023 was not high. The incidence of AEFI reports from the rotavirus vaccine is relatively low, indicating a favorable safety profile. Efforts should prioritize strengthening health education on rotavirus gastroenteritis to enhance public confidence in vaccination, thereby ensuring the effective prevention and control of rotavirus gastroenteritis.

## 1. Introduction

Rotavirus (RV) is the leading cause of severe gastroenteritis among children under five years of age. Infection may lead to serious complications such as severe dehydration, electrolyte imbalance, life-threatening diarrhea, and even mortality [1]. RV is primarily transmitted via the fecal–oral route, and infection can occur through contact with contaminated food, water, objects, or surfaces, making it highly contagious among children [2]. The epidemic peak typically occurs from October to February [3,4]. According to global data from 2016, RV infections were highly prevalent in children under five, with an incidence rate of 0.42 cases per person-year, resulting in approximately 18.882 million severe cases and 128,000 fatalities [5]. In China, surveillance from 2011 to 2018 indicated that rotavirus gastroenteritis (RVGE) accounted for 34.0% of diarrhea-related outpatient visits among children under five. The virus detection rate varied noticeably across different regions, with higher RV positivity rates in southern regions compared to northern areas during epidemic months [6] and higher rates in rural than in urban settings [7,8]. Surveillance data from the China Center for Disease Control and Prevention’s information system showed that the annual reported incidence of RVGE in China increased from 8.4 per 100,000 in 2005 to 178.1 per 100,000 in 2018, displaying a fluctuating upward trend [9]. Due to its high infectivity and multiple transmission routes, RV can cause outbreaks in settings such as households, childcare centers, and kindergartens.

Regarding the treatment of rotavirus infection, there are currently no effective antiviral drugs available. Consequently, vaccination is considered the most effective strategy for mitigating the disease burden and associated economic costs of rotavirus. The World Health Organization (WHO) recommends that rotavirus vaccines be regarded as an important component of the comprehensive strategy for controlling diarrheal diseases [10]. While global rotavirus vaccine coverage has been increasing annually, significant disparities exist across different regions [11]. For instance, the coverage rate in the United Kingdom is reported to be 87.4% and that in Canada is approximately 85% [12]. In China, the rotavirus vaccine is categorized as a non-National Immunization Program (non-NIP) vaccine, meaning it is voluntary and self-paid. As a result, the vaccination rate is relatively low and varies significantly among different regions. Currently, there are three main types of rotavirus vaccines available in the Chinese market: the rotavirus (live) vaccine, oral (LLR; Lanzhou Institute of Biological Products Co., Ltd., Lanzhou, China), the reassortant rotavirus vaccine, live, oral, pentavalent (RV5; Merck & Co., Inc. Rahway, NJ, USA), and rotavirus vaccine, live, oral, trivalent (LLR3; Lanzhou Institute of Biological Products Co., Ltd.). In Suzhou, LLR and RV5 are mainly used. LLR is suitable for children aged 2 months to 3 years, with one dose per year and an interval of 1 year between doses, with a maximum of three doses. RV5 is for infants aged 6 to 32 weeks, with a total of three doses. The first dose is given orally at 6 to 12 weeks of age, with an interval of 4 to 10 weeks between doses, and the third dose should not be later than 32 weeks of age.

Rotavirus vaccines have strict age restrictions and an early recommended vaccination schedule, making it easy to miss vaccinations. Compared with other vaccines, rotavirus vaccines differ in terms of vaccination methods, vaccination timelines, and prices, suggesting that the factors influencing their uptake may also be distinct. Additionally, most research in China focuses on non-NIP vaccines, which include rotavirus vaccines, rather than focusing specifically on rotavirus vaccines. There are relatively few specialized studies on rotavirus vaccines. This study analyzes the vaccination status and safety of rotavirus vaccines in a birth cohort of children born in Suzhou between 2017 and 2023. Evaluating the current status of rotavirus vaccination coverage in Suzhou and the safety profile of rotavirus vaccines is significant for improving vaccination willingness, protecting child health, and increasing socio-economic benefits.

## 2. Materials and Methods

### 2.1. Data Sources

Children registered in Suzhou City who were born between 1 January 2017 and 31 December 2023 were selected as the study cohort from the Jiangsu Province Immunization Service Management Information System. Personal information and RV vaccination information were collected. The data on individual cases of adverse events following immunization (AEFIs) associated with RV vaccine in the birth cohort from 2017 to 2023 were extracted from the AEFI monitoring system of the China Information System for Disease Control and Prevention, with the data export date being 31 July 2024.

### 2.2. Research Methods

Based on the National Surveillance Guideline for Adverse Events Following Immunization (AEFI) [13], all vaccines used post-marketing and under emergency authorization fall within the monitoring scope of the AEFI surveillance system under the China Information System for Disease Control and Prevention. Following an AEFI report from vaccination units or healthcare institutions, the county center for disease control and prevention is responsible for verification and investigation. Individual cases are then diagnosed and classified by AEFI investigation and diagnosis expert groups organized at the county, municipal, and provincial levels, according to their respective mandates and responsibilities. AEFIs encompass adverse reactions (including common reactions and abnormal reactions), vaccine quality-related incidents, immunization error-related incidents, coincidental events, and psychogenic reactions.

### 2.3. Calculation Formulas

RV vaccine proportion (%) = Number of children in the birth cohort who completed the specific vaccination/Total children in the annual birth cohort × 100%.

AEFI reporting rate = Number of reported AEFI cases for the vaccine/Total number of vaccine doses administered × 10,000.

In this study, the children in the annual birth cohort were defined as those registered in the Jiangsu Province Immunization Service Management Information System. For the purpose of this study, administration of the first dose of RV5 within the age range of 6 to 12 weeks was considered timely vaccination. At present, China mainly uses the vaccination rate of the National Immunization Program (NIP) to calculate vaccine coverage. For ease of comparison and description, the term “vaccine proportion” was used in this study. Its definition is derived with reference to the formula used to calculate the vaccine coverage.

### 2.4. Statistical Analysis

Data organization and statistical analysis were performed using WPS Office (version 12.1.0.24657), GraphPad Prism 8 and SPSS software (version 21.0). The delay time of first-dose RV5 was presented as median (M) with interquartile range (IQR). Between-group comparisons were analyzed using the Mann–Whitney U test for two independent cohorts, with statistical significance defined at *p* < 0.05.

## 3. Results

### 3.1. Study Population and Characteristics

The first-dose RV vaccine proportion was 14.65% for the birth cohort from 2017 to 2023 in Suzhou. And the full-course vaccine proportion was 3.81% for the birth cohort from 2017 to 2021. Regarding LLR, the vaccine proportions for the first-dose among children born in Suzhou from 2017 to 2023 were 8.59%. The first-dose vaccination proportion initially increased in earlier years followed by a subsequent decline. And the vaccine proportions for the second and third doses among children born from 2017 to 2021 were 4.56%, and 0.86%, respectively. For RV5, the vaccine proportions for the first, second, and third doses among children born in Suzhou from 2018 to 2023 were 6.06%, 6.13%, and 5.96%, respectively. The vaccine proportion for all three doses of RV5 demonstrated a gradual year-on-year increase, reaching the highest levels in the 2023 birth cohort, as shown in Table 1.

### 3.2. Distribution of Age for RV Vaccination

**Figure 1:**
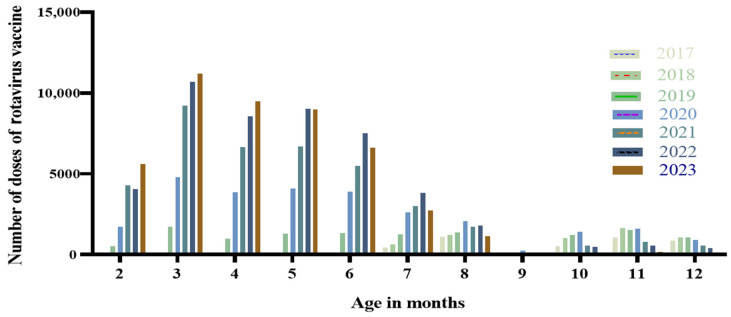
Distribution of RV vaccination age among children born in Suzhou from 2017 to 2023.

For the birth cohort of 2017–2023 in Suzhou, the peak age for RV vaccination was between 2 and 8 months. Among the birth cohorts from 2019 to 2023, the number of vaccine doses administered peaked at 3 months of age, and the proportions of doses administered at 3 months of age for these respective annual cohorts were 5.69%, 11.26%, 17.98%, 20.52%, and 24.00%, as shown in Figure 1.

### 3.3. Distribution of Delay in First Dose of RV5

Among the 2017–2023 birth cohort in Suzhou, 49,507 children received the first dose of RV5 between 6 and 12 weeks of age, achieving a timely vaccination rate of 99.88%. In contrast, only 57 children (0.12%) received the first dose after 12 weeks of age, and the median (interquartile range) of the first dose was 212 days (range: 194, 230). There was a statistically significant difference in the median extended duration of the first dose of RV5 among children of different age groups (*p* < 0.001), as shown in Table 2.

### 3.4. AEFIs from Rotavirus Vaccination

For the 2017–2023 birth cohort in Suzhou, a total of 89 cases of AEFIs were reported, corresponding to an overall AEFI reporting rate of 3.47 per 10,000 vaccine doses administered. A total of 86 cases were classified as general reactions (3.35/10,000), while three cases were identified as rare adverse reactions (0.12/10,000). Regarding gender, there were 44 males and 45 females. Furthermore, in terms of vaccination doses, 57 cases were reported for one dose, which accounted for the highest proportion, and the AEFI reporting rate was 4.76/10,000. Detailed data are presented in Table 3.

In terms of clinical diagnosis, general reactions included fever, diarrhea, and vomiting. There were 29 and 16 cases reported after LLR respectively, with AEFI reporting rates of 2.68/10,000 and 1.48/10,000. There were 18 (1.21/10,000) and 9 (0.61/10,000) cases reported after RV5, respectively. Regarding abnormal reactions, one case was reported after LLR, and two cases were reported after RV5. Detailed data are presented in Table 4.

## 4. Discussion

RVGE is a significant global public health threat to children’s health. Rotavirus infection is responsible for up to 200,000 child deaths annually [10]. Studies have indicated that among hospitalized pediatric cases of viral gastroenteritis, rotavirus-positive children account for 89.7%, with a predominance in children under 3 years of age in China [14]. Rotavirus infection contributes substantially to both outpatient and inpatient healthcare burdens. According to the study, the per capita costs for outpatients and inpatients caused by rotavirus were estimated to be RMB 389.85 and RMB 4131.10, respectively [15]. Vaccination is recognized as the most effective measure to mitigate the disease burden and economic burden associated with rotavirus gastroenteritis. The WHO recommends the inclusion of rotavirus vaccines into NIPs. However, significant disparities in vaccination coverage persist across different regions.

In recent years, the full-course vaccination rate for rotavirus vaccines among children in the Southeast Asia region has shown a notable increase, rising significantly to 68% in 2023 [16]. This upward trend may be associated with adjustments in immunization strategies in countries such as Vietnam and Thailand [17,18], which incorporated rotavirus vaccines into their national or regional immunization programs starting in 2020. In countries where rotavirus vaccines have not yet been included in the NIP, approximately 40% of diarrhea-related hospitalizations in children under 5 years of age are attributed to rotavirus infection [19]. Prior to the introduction of rotavirus vaccines in China, the hospitalization rate due to RV was 1100 per 100,000 children [20]. Following increased vaccine availability, studies reported that the annual incidence rates of RV-related outpatient visits and hospitalizations decreased to 2020 per 100,000 and 210 per 100,000, respectively [21]. Although the hospitalization and mortality rates associated with RV diarrhea have shown a declining trend, the disease burden of RVGE in China remains substantial. RVGE continues to be a noteworthy infectious disease in China, capable of causing fatal outcomes in children [22,23].

Some studies have indicated that the reported incidence of rotavirus diarrhea in children under 5 years of age was 178.1 per 100,000 from 2005 to 2018 [9]. Regional studies have further demonstrated that rotavirus is a leading pathogen responsible for viral diarrhea among children under 5 years old in Suzhou [24]. Despite its clinical significance, rotavirus vaccination remains a non-NIP vaccine in China, and overall vaccination coverage remains relatively low [25]. This study analyzed the data of children born in Suzhou from 2017 to 2023, aiming to provide a detailed local vaccination pattern and safety profile. The first-dose vaccination proportion of the rotavirus vaccine among children born in Suzhou from 2017 to 2023 was 14.65%. These rates are notably lower than those reported in other regions, such as Kaifeng (35.40%) [26] and Beijing (35.93%) [27]. Possible reasons include the self-funded vaccination policy and the insufficient awareness of parents about RVGE and rotavirus vaccines [28,29]. The study revealed that after 2020, the vaccination proportion for LLR gradually declined, whereas the proportion for RV5 demonstrated a steady increase. This trend is consistent with observations from Kaifeng [26]. From a market share perspective, the proportion of LLR utilized has progressively decreased, while that of RV5 has risen. This shift may be attributed to the earlier recommended age for the first dose of RV5 and the relatively shorter timeframe required to complete its full vaccination course. If the immunogenicity of the vaccine is maintained, an immunization schedule with shorter intervals between doses may be more conducive to improving full-course vaccination coverage.

This study found that the peak age for rotavirus vaccination was between 2 and 8 months. In Suzhou, the rotavirus detection rate was highest among children aged 19 to 24 months, followed by the age group from 13 to 18 months. Generally, antibody concentrations peak about four weeks after vaccination, providing robust protection. In a large-scale clinical trial conducted across several Latin American countries, the studied vaccine remained effective in preventing approximately 80% of severe rotavirus gastroenteritis cases and 83% of related hospitalizations within two years post-vaccination [30]. Another study showed that the adjusted protective efficacy of the oral live rotavirus vaccine in children aged 2 to 35 months was 36.2% [31], and the protective effect strengthened with an increasing number of doses administered [32]. Therefore, timely vaccination within the recommended timeframe can provide enhanced protection for children.

LLR is suitable for infants and young children from 2 months to 32 months of age. Consequently, for those who have missed the recommended window for the first dose of RV5, LLR presents a preferable alternative. This explains why the administration of the first dose of LLR is predominantly concentrated after 32 weeks of age. Existing research has also identified that the vaccination schedule for LLR is often between 5 to 9 months of age, which is consistent with the findings of this study [33]. For RV5, the recommended schedule for the first dose is between 6 to 12 weeks of age, and the majority of children receive vaccinations within this period. Delaying vaccination postpones the onset of the vaccine’s protective effect, thereby increasing the risk of rotavirus infection in infants. Therefore, it is advised that infants receive their first dose of the rotavirus vaccine as early as possible after reaching 6 weeks of age and complete the full vaccination course. Research indicates that the earlier administration of LLR yields a better protective effect. Specifically, the vaccine efficacy rate reaches 90.00% for those who receive the first dose between 2 to 6 months of age, which is significantly higher than the 73.00% efficacy observed for those starting vaccination between 7 to 11 months of age [34]. This underscores that initiating vaccination as soon as the infant reaches the minimum eligible age stipulated by the immunization schedule is crucial for optimal protection. If the first dose is administered later, the completion of the full course will also be delayed, potentially preventing the vaccine from conferring its maximum protective benefit. Hence, it is recommended to carefully coordinate the timing of both NIP vaccines and non-NIP vaccines to ensure the rotavirus vaccination series is completed at the earliest opportunity.

The reported incidence of AEFIs for the 2017–2023 birth cohort in Suzhou was 3.47 per 10,000 doses. This rate is lower than that reported for the 2019–2023 birth cohort in Kaifeng (6.34 per 10,000 doses) [26] but higher than the rate of 25.58 per 100,000 doses documented in Jiangsu province from 2019 to 2022 [35]. These observed differences may be attributed to variations in the statistical methodologies used to define the birth cohort, the criteria for counting AEFI cases, and the calculation of total vaccine doses administered across the studies. The higher reported incidence of the first dose may be attributed to a stronger first immune response and more doses [36]. The findings indicate a relatively low level of abnormal reactions. The majority of reported events were common reactions, predominantly presenting as transient symptoms such as fever and diarrhea, which suggests a favorable safety profile for the rotavirus vaccine in Suzhou.

This study has several limitations. First, the estimated number of children in the annual birth cohort was derived from children registered within the Jiangsu Provincial Comprehensive Service Management Information System for Vaccination. Although this system provides a robust estimation of the registered child population, the potential influence of population mobility (e.g., the floating population) cannot be entirely ruled out, which might introduce slight deviations in coverage estimates. Future research can construct a more stable birth cohort through multiple databases such as household registration management and maternal and child healthcare in order to reduce biases. Second, the AEFI data were primarily collected through a passive surveillance system, which is potentially susceptible to underreporting and incomplete data, both of which could affect the accuracy of the vaccine safety assessment. In the future, a combined approach of passive monitoring and active monitoring can be adopted (such as conducting prospective studies in specific populations or sentinel hospitals) to obtain more accurate and comprehensive data on the incidence of AEFIs, thereby enabling a more comprehensive assessment of vaccine safety.

## 5. Conclusions

In conclusion, the rotavirus vaccine coverage in Suzhou remains suboptimal. Given the demonstrated favorable safety profile of the rotavirus vaccine, it is recommended to strengthen specialized health education focused on RVGE. Efforts should aim to enhance parents’ awareness of rotavirus infection, to improve vaccination confidence, and to ensure the effective prevention of rotavirus gastroenteritis.

## Figures and Tables

**Table 1:** Information on RV vaccination of children born in Suzhou from 2017 to 2023.

Birth Year	Total Number of Children (*N*)	LLR			RV5			First Dose of Vaccination *n* (%)	Full Course Vaccination *n* (%)
		LLR_1_ *n* (%)	LLR_2_ *n* (%)	LLR_3_ *n* (%)	RV5_1_ *n* (%)	RV5_2_ *n* (%)	RV5_3_ *n* (%)		
2017	157,730	11,971 (7.59%)	2602 (1.65%)	103 (0.07%)	-	-	-	11,971 (7.59%)	103 (0.07%)
2018	134,988	13,872 (10.28%)	4194 (3.11%)	623 (0.46%)	8 (0.01%)	4 (0.00%)	5 (0.00%)	13,880 (10.28%)	628 (0.47%)
2019	136,623	15,476 (11.33%)	8094 (5.92%)	1193 (0.87%)	2201 (1.61%)	2078 (1.52%)	1642 (1.20%)	17,677 (12.94%)	2835 (2.08%)
2020	112,374	13,124 (11.68%)	8043 (7.16%)	1966 (1.75%)	6009 (5.35%)	6485 (5.77%)	6845 (6.09%)	19,133 (17.03%)	8811 (7.84%)
2021	101,172	8497 (8.40%)	6414 (6.34%)	1630 (1.61%)	12,419 (12.28%)	11,739 (11.60%)	10,490 (10.37%)	20,916 (20.67%)	12,120 (11.98%)
2022	91,492	5499 (6.01%)	NA	NA	13,802 (15.09%)	14,741 (16.11%)	15,086 (16.49%)	19,301 (21.10%)	NA
2023	82,901	1744 (2.10%)	NA	NA	15,125 (18.24%)	15,044 (18.15%)	14,645 (17.67%)	16,869 (20.35%)	NA
Total	817,280	70,183 (8.59%)	29,347 * (4.56%)	5515 * (0.86%)	49,564 (6.06%)	50,091 (6.13%)	48,713 (5.96%)	119,747 (14.65%)	24,497 * (3.81%)

Notes: Data are presented as *n* (%). *N* represents the total number of children for each birth year, and *n* represents the number of children in the birth cohort who received the specific vaccination. “First dose of vaccination” denotes receipt of one dose of either LLR or RV5. “Full course vaccination” denotes completion of the three-dose series for either vaccine. “-” indicates zero. “NA” indicates data are not available or not applicable (e.g., incomplete follow-up for later doses in recent birth cohorts). *: The statistical scope of “LLR_2_”, “LLR_3_”, and “full course vaccination” was the birth cohort from 2017 to 2021.

**Table 2:** Distribution of delay in first dose of RV5 among children born in Suzhou from 2017 to 2023.

Vaccination Age (Weeks)	First-Dose RV5		
	Number of Children Vaccinated	Proportion (%)	Duration, Day [M (P25, P75)]
6–12	49,507	99.88	64 (43,85)
>12	57	0.12	212 (194,230)

Notes: M indicates median; P25 and P75 indicate the 25th and 75th percentiles, respectively. The duration day between the two groups was compared using the Mann–Whitney U test (Z = 13.07, *p* < 0.001).

**Table 3:** Reported incidence rates of AEFIs among children born in Suzhou from 2017 to 2023.

Variable	2017 *n* (Rate)	2018 *n* (Rate)	2019 *n* (Rate)	2020 *n* (Rate)	2021 *n* (Rate)	2022 *n* (Rate)	2023 *n* (Rate)	Total	
								Cases	Incidence Rate *(95% CI)*
Classification									
General reaction	6 (4.09)	7 (3.74)	19 (6.19)	11 (2.59)	17 (3.32)	13 (2.49)	13 (2.78)	86	3.35 (2.64–4.06)
Rare adverse reaction	0 (0.00)	0 (0.00)	0 (0.00)	2 (0.47)	1 (0.20)	0 (0.00)	0 (0.00)	3	0.12 (0.02–0.34)
Gender									
Male	3 (3.95)	5 (5.14)	8 (5.01)	6 (2.78)	7 (2.67)	6 (2.25)	9 (3.77)	44	3.34 (2.36–4.33)
Female	3 (4.24)	2 (2.23)	11 (7.48)	7 (3.35)	11 (4.41)	7 (2.74)	4 (1.75)	45	3.60 (2.55–4.65)
Dose									
1	6 (5.01)	5 (3.60)	10 (5.66)	10 (5.23)	11 (5.26)	6 (3.11)	9 (5.34)	57	4.76 (3.52–5.99)
2	0 (0.00)	2 (4.76)	7 (6.88)	0 (0.00)	4 (2.20)	4 (2.26)	4 (2.64)	21	2.55 (1.46–3.63)
3	0 (0.00)	0 (0.00)	2 (7.05)	3 (3.40)	3 (2.48)	3 (1.98)	0 (0.00)	11	2.03 (0.83–3.22)
Total	6 (4.09)	7 (3.74)	19 (6.19)	13 (3.06)	18 (3.52)	13 (2.49)	13 (2.78)	89	3.47 (2.75–4.19)

Notes: Data for 2017–2023 are presented as number of cases (incidence rate), *n* represents the number of reported AEFI cases. The incidence rate is calculated per 10,000 doses. The “Total” column presents the incidence rate with its 95% confidence interval (CI).

**Table 4:** Distribution of RV adverse reactions among children born in Suzhou from 2017 to 2023.

Clinical Diagnosis	LLR		RV5		Total	
	Cases	Incidence Rate (/10,000 Doses)	Cases	Incidence Rate (/10,000 Doses)	Cases	Incidence Rate (/10,000 Doses)
General reaction						
Fever	29	2.68	18	1.21	47	1.83
Redness and swelling	1	0.09	0	0.00	1	0.04
Induration	1	0.09	0	0.00	1	0.04
Diarrhea, vomiting	16	1.48	9	0.61	25	0.97
Fatigue, drowsiness	5	0.46	9	0.61	14	0.55
Loss of appetite	6	0.55	2	0.13	8	0.31
Rash	2	0.18	2	0.13	4	0.16
Cough	0	0.00	2	0.13	2	0.08
Abnormal reaction						
Thrombocytopenic purpura	0	0.00	1	0.07	1	0.04
Other diseases	1	0.09	1	0.07	2	0.08

## Data Availability

The data are not publicly available due to the sensitive nature of the information. The data will be made available from the corresponding author upon request.
